# Phenotypic and biochemical characterization of the class C β-lactamase, PAC-1

**DOI:** 10.1093/jac/dkag291

**Published:** 2026-08-25

**Authors:** Otávio Hallal Ferreira Raro, Stefano Mancini, Jacqueline Findlay, Helena M B Seth-Smith, Muhammad Ali Syed, Adrian Egli, Patrice Nordmann, Laurent Poirel

**Affiliations:** Medical and Molecular Microbiology Unit—Department of Oncology, Microbiology, Immunology (OMI), Faculty of Science and Medicine, University of Fribourg, Chemin du Musée 18, Fribourg CH-1700, Switzerland; Swiss National Reference Center for Emerging Antibiotic Resistance (NARA), Faculty of Science and Medicine, University of Fribourg, Fribourg, Switzerland; Institute of Medical Microbiology, University of Zurich, Zurich, Switzerland; Medical and Molecular Microbiology Unit—Department of Oncology, Microbiology, Immunology (OMI), Faculty of Science and Medicine, University of Fribourg, Chemin du Musée 18, Fribourg CH-1700, Switzerland; Institute of Medical Microbiology, University of Zurich, Zurich, Switzerland; SIB Swiss Institute of Bioinformatics, Zurich, Switzerland; Department of Microbiology, The University of Haripur, Haripur, Pakistan; Institute of Medical Microbiology, University of Zurich, Zurich, Switzerland; Medical and Molecular Microbiology Unit—Department of Oncology, Microbiology, Immunology (OMI), Faculty of Science and Medicine, University of Fribourg, Chemin du Musée 18, Fribourg CH-1700, Switzerland; Swiss National Reference Center for Emerging Antibiotic Resistance (NARA), Faculty of Science and Medicine, University of Fribourg, Fribourg, Switzerland; Medical and Molecular Microbiology Unit—Department of Oncology, Microbiology, Immunology (OMI), Faculty of Science and Medicine, University of Fribourg, Chemin du Musée 18, Fribourg CH-1700, Switzerland; Swiss National Reference Center for Emerging Antibiotic Resistance (NARA), Faculty of Science and Medicine, University of Fribourg, Fribourg, Switzerland

## Abstract

**Background:**

The class C β-lactamase, PAC-1, was originally described as acquired in *Pseudomonas aeruginosa* clinical isolates from south Asia, the corresponding gene being chromosomally located and embedded within a Tn*1721*-like transposon. Despite its association with high-level resistance to cephalosporins and β-lactam/β-lactamase inhibitor combinations, data on its full hydrolytic profile and kinetic parameters have not yet been comprehensively characterized.

**Objectives:**

To characterize the susceptibility profile and hydrolytic kinetics of PAC-1, with a particular focus on cefiderocol hydrolysis, and the activity of novel β-lactam/β-lactamase inhibitor combinations.

**Methods:**

The *bla*_PAC-1_ allele was cloned into vector pUCP24 and expressed in *Escherichia coli* TOP10 and *Pseudomonas aeruginosa* PAO1, respectively. MICs were determined by broth microdilution and interpreted according to EUCAST guidelines. PAC-1 was purified, and steady-state kinetic measurements of selected β-lactams were determined. IC_50_ values were determined for the β-lactamase inhibitors avibactam, taniborbactam, xeruborbactam and zidebactam. WGS was performed to characterize the genetic environment of the *bla*_PAC-1_ gene.

**Results:**

PAC-1 conferred broad resistance to penicillins, broad-spectrum cephalosporins and cefiderocol, while carbapenems remained unaffected. Kinetic analyses confirmed high catalytic efficiency against cefotaxime and aztreonam, but no hydrolysis of imipenem. Avibactam, relebactam and vaborbactam failed to inhibit PAC-1 (IC_50_ > 1000 µM), whereas xeruborbactam and zidebactam showed potent inhibitory activities (IC_50_ = 1.0 and 0.6 µM, respectively). A chromosomally located Tn*1721*-like transposon was identified carrying the *bla*_PAC-1_ gene in *P. aeruginosa* ST4936.

**Conclusions:**

PAC-1 is not inhibited by avibactam and hydrolyses a broad range of β-lactams including the novel cephalosporin cefiderocol. The combination cefiderocol/zidebactam was the most active against PAC-1. Transposon-mediated dissemination across distinct lineages warrants continued epidemiological surveillance of PAC-type β-lactamases.

## Introduction

The acquired *bla*_PAC-1_ Ambler class C β-lactamase gene was first described in a *Pseudomonas aeruginosa* clinical strain isolated from a patient hospitalized in India in 2010.^1^ Its circulation remains rare, and reports have been specific to *P. aeruginosa* ST664 isolates of both clinical and animal origins recovered from the south Asia region (India, Nepal, Singapore), France (from patients repatriated from Afghanistan and Mauritius), as well as environmental isolates of *Aeromonas caviae* recovered from water reservoirs.^1–5^ PAC-1 cephalosporinase shares 47% amino acid identity with the intrinsic chromosomal PDC-1 β-lactamases, suggesting a distinct evolutionary origin.^3^ The *bla*_PAC-1_ gene was always identified as chromosomally located, embedded within a Tn*1721*-like transposon.^3,5^ The first description of a *P. aeruginosa* lineage, other than ST664, producing the PAC-1 β-lactamase was from a group in the USA.^6^ This strain is ST4936 and co-produces PAC-1 and the MBL imipenemase-1 (IMP-1) enzymes, a combination recently reported in *P. aeruginosa* clinical isolates responsible for an outbreak in Pakistan.^7^ Apart from the Americas and Asia, a PAC-1-producing *P. aeruginosa* veterinary isolate assigned to ST307 was identified also in Greece.^8^

A variant of PAC-1 exhibiting three amino acid substitutions (K44Q, E309A and N339K) and therefore named PAC-2 (PQ567105.1) was recently identified in a *Klebsiella quasipneumoniae* clinical isolate recovered in Colombia.^9^ The *bla*_PAC-2_ gene was further reported in seven Enterobacterales clinical isolates in Colombia, including *Klebsiella pneumoniae*, *Kluyvera ascorbata*, *Enterobacter hormaechei*, *Serratia marcescens* (co-producing *bla*_KPC-2_) and *Enterobacter asburiae* (co-producing *bla*_KPC-2_).^10^ Genomic analyses identified that the *bla*_PAC-2_ gene was located on a 9.3 kb IncQ-type plasmid in all seven isolates. This raised the concern of whether PAC-type enzymes had already disseminated globally.^10^

PAC-1 β-lactamase confers resistance to penicillins and to a wide range of cephalosporins, including ceftazidime, cefepime, ceftolozane/tazobactam and ceftazidime/avibactam when produced in *P. aeruginosa*.^3,11^ Previous studies showed that the expression of *bla*_PAC-1_ in *Escherichia coli* TOP10 and *P. aeruginosa* PAO1 strain backgrounds did not impact carbapenem susceptibility.^3^ Mancini *et al.*^7^ demonstrated that production of PAC-1 in a recombinant *E. coli* strain resulted in a 5-fold increase of cefiderocol MICs. However, there are currently no data regarding the hydrolytic profile of PAC-1 against a wide range of antibiotics, including β-lactam/β-lactamase inhibitors. Therefore, the aim of the present study was to determine the hydrolytic features of PAC-1, its impact on cefiderocol susceptibility, and to evaluate precisely the activity of different β-lactamase inhibitors against that enzyme.

## Materials and methods

### Bacterial strain and bla_PAC-1_ construct

A clinical *P. aeruginosa* ST4936 strain (named PaCS), isolated in 2025 from a patient hospitalized at the Khyber Teaching Hospital, Peshawar, Pakistan and carrying the *bla*_PAC-1_ gene, was selected for performing the cloning experiment.^7^ Cloning was performed into shuttle vector pUCP24 (pUCP24-PAC1) to enable expression in both *E. coli* TOP10 and *P. aeruginosa* PAO1 strain backgrounds.^12^ The *bla*_PAC-1_ gene was amplified by PCR and cloned into vector using primers PAC-1_F 5′-GAT GAT GGA TCC TAT AGA ATT CAA CAC GTC TCT ACC CTG AAT G-3′ and PAC-1_R 5′-GAT GAT AAG CTT TAT AGG ATC CTC TCT ATG CGG TTG GCT GTG-3′, before transforming into *E. coli* TOP10. Clones were selected on LB agar plates supplemented with 15 mg/L gentamicin and confirmed by amplification and sequencing of the *bla*_PAC-1_ gene. The recombinant plasmid pUCP24-PAC-1 was then transformed by electroporation into *P. aeruginosa* strain PAO1.

### Antimicrobial susceptibility testing

MICs were determined by broth microdilution using CAMHB (Bio-Rad, Marnes-la-Coquette, France) for all the antibiotics except for cefiderocol, for which testing was performed using an iron-depleted CAMHB, according to EUCAST guidelines.^13^ Iron-depleted CAMHB was also used when testing combinations of cefiderocol with the β-lactamase inhibitors avibactam, relebactam, vaborbactam, taniborbactam, xeruborbactam and zidebactam. The β-lactamase inhibitors were all tested at fixed concentrations: clavulanic acid at 2 mg/L; tazobactam, avibactam, relebactam, taniborbactam and zidebactam at 4 mg/L; and vaborbactam and xeruborbactam at 8 mg/L. *E. coli* ATCC 25922, *P. aeruginosa* ATCC 27853, and *K. pneumoniae* strains 700603 and BAA-2817 were used as controls.

### Enzyme purification

For the purpose of optimal purification, the *bla*_PAC-1_ gene was cloned into the expression plasmid pOPINF (pOPINF-PAC-1) by using the In-Fusion^™^ cloning kit (Takara Bio, Japan).^14^ The primers used for cloning were designed without the signal peptide (as predicted by SignalP-5.0; https://services.healthtech.dtu.dk/services/SignalP-5.0/) and for generating the enzyme with an N-terminal His-tag. Primers were the following: PAC-1_pOPINF_F 5′-GAA GTT CTG TTT CAG GGT CAA GTA TCA CAA GCC GAT-3′ and PAC-1_pOPINF_R 5′-TGG TCT AGA AAG CTC TAT TTT TTC AGT CGT TGT ACC A-3′. Recombinant plasmid pOPINF-PAC-1 was then transformed into strain BL21 DE3 (pLysS) (BL21-DE3-pOPINF-PAC-1). Transformants were selected on LB agar plates supplemented with 100 mg/L ampicillin, and successful transformants were confirmed by sequencing of the pOPINF-PAC-1 plasmid.

Expression of the *bla*_PAC-1_ gene cloned into BL21-DE3-pOPINF-PAC-1 was induced with 0.5 mM/L IPTG (Carl Roth, Germany). PAC-1 was then extracted by sonication, and supernatant was purified by a nickel-nitrilotriacetic acid (Ni-NTA) agarose column (His GraviTrap^TM^; GE Healthcare, USA) as previously described.^15^ Purified protein was analysed by SDS-PAGE.

### Determination of kinetic parameters

Steady-state kinetic assays were performed for the purified PAC-1 enzyme, as described elsewhere.^16^ Hydrolysis of nitrocefin, piperacillin, cefotaxime, ceftazidime, cefepime, imipenem, cefiderocol, aztreonam, cephalotin and benzylpenicillin were observed by the reduction of absorbance using SpectraMax ID5 equipment (Molecular Devices, San Jose, CA, USA). Experiments were performed in 50 mM Tris-HCl and 300 mM NaCl buffer. Absorbances were measured for seven different substrate concentrations for 30 min at 25°C. Data were analysed in GraphPad Prism v9.5.1 (GraphPad Prism Software, Boston, MA, USA) using a non-linear regression curve according to Michaelis–Menten. Antibiotic coefficients are the following: nitrocefin (Δɛ486 = 15 900 M^−1^ cm^−1^), piperacillin (Δɛ235 = 1070 M^−1^ cm^−1^), cefotaxime (Δɛ264 = −7250 M^−1^ cm^−1^), ceftazidime (Δɛ260 = −8860 M^−1^ cm^−1^), cefepime (Δɛ267 = −9120 M^−1^ cm^−1^), imipenem (Δɛ297 = 9210 M^−1^ cm^−1^), cefiderocol (Δɛ259 = −9430 M^−1^ cm^−1^), aztreonam (Δɛ318 = −650 M^−1^ cm^−1^), cephalotin (Δɛ262 = −7660 M^−1^ cm^−1^) and penicillin G (Δɛ233 = −775 M^−1^ cm^−1^). All experiments were performed in triplicate.

### Determination of the IC_50_

PAC-1 enzyme was incubated with avibactam, relebactam, vaborbactam, taniborbactam, xeruborbactam or zidebactam for 3 min at room temperature in 100 mM sodium phosphate buffer (pH 7.0). After preincubation time, the hydrolytic activity of PAC-1 was obtained by measuring the hydrolysis rate of cephalotin (100 μM) at 262 nm as previously described.^17^

### WGS

WGS was performed on a NextSeq 1000 instrument (Illumina; https://www.illumina.com) with 2 × 150 bp paired-end reads following QiaSeq FX (Qiagen) library preparation. For long-read (Nanopore) sequencing, DNA was extracted using QIAamp DNA minikit (Qiagen, Valencia, CA, USA) and sent to Microsynth (https://srvweb.microsynth.ch/) where long-read sequencing was performed using Oxford Nanopore Technologies (ONT) on a MinION flow cell (FLO-MIN114; ONT, Oxford, UK). Hybrid assemblies, using long and short reads, were performed using UniCycler.^18^ Sequencing reads were mapped to the Tn*1721*-like transposon originally found in *P. aeruginosa* (accession number: MK534438) by using the CLC Genomics Workbench 20.0.4 Qiagen Aarhus A/S (Qiagen, Redwood City, CA, USA). Sequencing datasets from this study were deposited in the NCBI Sequence Read Archive under BioProject accession number PRJNA1431343.

## Results and discussion

### Phenotypic profiling of recombinant E. coli and P. aeruginosa strains producing PAC-1

The expression of the *bla*_PAC-1_ gene in *E. coli* TOP10 resulted in a broad resistance phenotype affecting all β-lactam classes tested, apart from the carbapenems (meropenem and imipenem) (Table 1). The *E. coli* TOP10 recombinant strain producing PAC-1 was resistant to penicillins and penicillin/β-lactamase inhibitor combinations. It was also resistant to broad-spectrum cephalosporins, including cefotaxime (MIC >128 mg/L), ceftazidime and ceftazidime/avibactam (MICs 512 mg/L), and cefepime (MIC 64 mg/L). The MICs observed using the combinations cefepime/avibactam, cefepime/taniborbactam and cefepime/zidebactam were, respectively, 8, 4 and ≤0.125 mg/L for the TOP10 recombinant strain producing PAC-1.

**Table 1.** Phenotypic profile of TOP10 and PAO1 producing PAC-1

Compound	MICs for strains, mg/L				
	TOP10	TOP10-pUCP24-PAC-1	PAO1	PAO1-pUCP24-PAC-1	PaCS
AMP	2	64	>128	>128	>128
AMX	8	>128	>128	>128	>128
AMC	4	>128	>128	>128	>128
PIP	1	16	4	>128	>128
TZP	1	16	4	>128	>128
TIC	2	>128	8	>128	>128
TIM	2	>128	16	>128	>128
CTX	≤0.125	>128	>128	>128	>128
CAZ	0.5	512	1	>1024	>1024
CZA	0.25	512	1	>1024	>1024
FEP	≤0.125	64	0.5	1024	1024
FPA	≤0.125	8	0.5	1024	1024
FTN	≤0.125	4	0.5	1024	1024
FEZ	≤0.125	≤0.125	≤0.125	≤0.125	512
IPM	0.25	0.25	1	2	128
IMR	0.25	0.25	0.5	2	128
MEM	≤0.125	≤0.125	1	2	128
MVB	≤0.125	≤0.125	0.5	2	128
FDC	≤0.125	4	0.5	>1024	64
FAV	≤0.125	4	0.5	>1024	64
FRL	≤0.125	4	0.5	>1024	64
FVB	≤0.125	4	0.5	>1024	64
FTB	≤0.125	1	0.5	32	16
FXB	≤0.125	≤0.125	0.5	16	8
FZB	≤0.125	≤0.125	≤0.125	≤0.125	2
ZID	≤0.125	≤0.125	2	2	4
ATM	≤0.125	4	8	32	32
AZA	≤0.125	4	8	32	32
FOF	0.5	0.5	>128	>128	>128
AMK	2	2	4	4	4
CXM	4	>128	>128	>128	>128
C/T	0.25	64	0.25	>128	>128

AMC, amoxicillin/clavulanic acid; AMK, amikacin; AMP, ampicillin; AMX, amoxicillin; ATM, aztreonam; AZA, aztreonam/avibactam; C/T, ceftolozane/tazobactam; CAZ, ceftazidime; CTX, cefotaxime; CXM, cefuroxime; CZA, ceftazidime/avibactam; FAV, cefiderocol/avibactam; FDC, cefiderocol; FEP, cefepime; FEZ, cefepime/zidebactam; FOF, fosfomycin; FPA, cefepime/avibactam; FRL, cefiderocol/relebactam; FTN, cefepime/taniborbactam; FVB, cefiderocol/vaborbactam; FTB, cefiderocol/taniborbactam; FXB, cefiderocol/xeruborbactam; FZB, cefiderocol/zidebactam; IMR, imipenem/relebactam; IPM, imipenem; MEM, meropenem; MVB, meropenem/vaborbactam; PaCS, *Pseudomonas aeruginosa* clinical strain; PIP, piperacillin; TIC, ticarcillin; TIM, ticarcillin/clavulanic acid; TZP, piperacillin/tazobactam; ZID, zidebactam.

Notably, resistance to cefiderocol was also observed, with an MIC of 4 mg/L while the MIC of the recipient TOP10 strain is <0.125 mg/L. When combining cefiderocol with avibactam, relebactam, vaborbactam or taniborbactam, no significant impact was observed on the MICs, suggesting that these inhibitors are not effective against PAC-1, despite the fact that avibactam is known to be effective against class C enzymes. In contrast, when combining cefiderocol with xeruborbactam or zidebactam, significantly lower MICs were observed, restoring the same susceptible values of MICs observed for the recipient TOP10 strain (<0.125 mg/L).

In *P. aeruginosa* PAO1, expression of *bla*_PAC-1_ led to high MICs of ceftazidime, ceftazidime/avibactam, cefepime, cefepime/avibactam, cefepime/taniborbactam, cefiderocol, cefiderocol/avibactam, cefiderocol/relebactam and cefiderocol/vaborbactam, all exceeding 1024 mg/L. By contrast, production of PAC-1 did not have any significant impact on imipenem and meropenem MICs. Altogether, these results illustrate that the production of PAC-1 in *P. aeruginosa* considerably narrows the available therapeutic options for infections caused by such isolates.

Among the β-lactam/β-lactamase inhibitor combinations evaluated, cefepime/zidebactam and cefiderocol/zidebactam were the only ones to retain full activity in a PAO1 recombinant strain producing PAC-1 (MIC ≤ 0.125 mg/L), the same result as observed for the TOP10 strain producing PAC-1, indicating effective inhibitor-mediated suppression of PAC-1 activity. Notably, the combination cefepime/zidebactam was inactive against the IMP1-producing PaCS isolate (MIC 512 mg/L), whereas cefiderocol/zidebactam retained good activity (MIC 2 mg/L). By analysing PaCS genomic data we also observed a truncation in the OprD membrane porin that is probably contributing, together with the expression of PAC-1, to the high level of resistance to carbapenems (especially imipenem) in the clinical strain. These results position cefiderocol/zidebactam as a potentially useful alternative against PAC-1-producing isolates and warrant further investigation in more complex resistance backgrounds (Table 1).

### Enzyme kinetic parameters of PAC-1

Enzymatic kinetic parameters of PAC-1 were determined against a panel of β-lactams (Table 2). Amongst the β-lactams tested, cefotaxime was the most efficiently hydrolysed substrate (*k*_cat_/*K*_m_ = 1.29 × 10^5^ M^−1^ s^−1^). This agrees fully with the phenotypic data, where cefotaxime MICs increased from ≤0.125 to >128 mg/L upon PAC-1 production. Hydrolysis of cefepime and ceftazidime was also observed (*k*_cat_/*K*_m_ = 6.57 × 10^4^ and 3.25 × 10^4^ M^−1^ s^−1^, respectively), consistent with the pronounced MIC elevations observed for these compounds in both TOP10 and PAO1 backgrounds. Lower catalytic efficiencies were observed against piperacillin and benzylpenicillin (*k*_cat_/*K*_m_ = 1.32 × 10^4^ and 2.65 × 10^4^ M^−1^ s^−1^), yet the corresponding MIC increases still reached clinically significant levels, which could be attributed to the contribution of enzyme production levels and cellular context to the final resistance phenotype. Notably, imipenem yielded no detectable hydrolysis under the conditions tested, confirming the absence of carbapenemase activity in PAC-1, in line with the MIC data.

**Table 2.** Kinetic parameters and IC_50_ values for PAC-1 enzyme against several β-lactams and β-lactam/β-lactamase inhibitors

Substrate	Kinetics		
	*K*_m_ (μM)	*k*_cat_ (s^−1^)	*k*_cat_/*K*_m_ (M^−1^ s^−1^)
NCF	175.6	32.1	1.83E + 05
PIP	345.5	4.57	1.32E + 04
CTX	35.2	4.55	1.29E + 05
CAZ	155.3	5.05	3.25E + 04
FEP	81.1	5.32	6.57E + 04
IPM^a^	—	—	—
FDC	141.0	1.70	1.21E + 04
ATM	4.4	0.48	1.10E + 05
CEF	19.9	0.78	3.96E + 04
PEN	58.9	1.56	2.65E + 04
**Inhibitor**	**IC_50_ (µM)**		
AVI	>1000		
REL	>1000		
VAB	>1000		
TNB	38.5		
XBT	1.0		
ZID	0.6		

ATM, aztreonam; AVI, avibactam; CAZ, ceftazidime; CEF, cephalotin; CTX, cefotaxime; FDC, cefiderocol; FEP, cefepime; IPM, imipenem; NCF, nitrocefin; PEN, penicillin G; PIP, piperacillin; REL, relebactam; TNB, taniborbactam; VAB, vaborbactam; XBT, xeruborbactam, ZID, zidebactam.

^a^Dash denotes no significant hydrolysis.

Among novel β-lactamase inhibitors, diazabicyclooctanes (DBOs) and boronic acid–based compounds generally retain activity against class C enzymes. However, avibactam failed to inhibit PAC-1 at concentrations higher than 1000 µM, which directly accounts for the lack of significant activity of ceftazidime/avibactam or cefiderocol/avibactam compared with ceftazidime alone when considering the MIC data. The same lack of significant activity was observed for imipenem/relebactam, cefiderocol/relebactam, meropenem/vaborbactam and cefiderocol/vaborbactam compared with imipenem, meropenem and cefiderocol alone, consistent with IC_50_ values for relebactam and vaborbactam exceeding 1000 µM. In contrast, zidebactam proved to be a potent inhibitor of PAC-1 (IC_50_ = 0.6 µM), and xeruborbactam showed comparable potency (IC_50_ = 1.0 µM), while taniborbactam displayed moderate inhibitory activity (IC_50_ = 38.5 µM). Notably, the MIC reductions observed for cefiderocol/zidebactam against PAC-1-producing strains were more pronounced than those of cefiderocol/xeruborbactam relative to cefiderocol alone, consistent with the comparable inhibitory potency of xeruborbactam and zidebactam. This finding likely reflects the dual mechanism of action of zidebactam, which apart from class A and C serine β-lactamase inhibition, exhibits direct antibacterial activity through high-affinity binding to PBP-2, providing an additional mechanism of action independent of enzymatic inhibition.^19^ Taken together, these kinetic data not only validate the resistance phenotype observed but also highlight the mechanistic distinction between inhibitor scaffolds: while avibactam-based combinations are rendered ineffective by PAC-1, zidebactam-containing regimens retain efficacy through potent inhibition of the enzyme, positioning cefiderocol/zidebactam as a particularly promising therapeutic option against PAC-1-producing pathogens.

### Analysis of the PAC-1 genetic environment

WGS analysis revealed that the *bla*_PAC-1_ gene was chromosomally located within a Tn*1721*-like transposon identical to the one already reported by Bour *et al*.^3^ Previous reports specifically associated the Tn*1721*-like carrying *bla*_PAC-1_ with ST664, and the detection of an identical element in ST4936 in this study suggests that dissemination of this resistance determinant can occur through transposition events across distinct lineages, independent of clonal expansion or plasmid-mediated transfer. This underscores the role of mobile elements in the spread of novel β-lactamases in clinical settings.

### Conclusions

This study provides a comprehensive phenotypic and biochemical characterization of the class C β-lactamase, PAC-1, confirming its broad hydrolytic spectrum against extended-spectrum cephalosporins and, notably, cefiderocol. This is particularly concerning, as the latter agent represents an important last-resort therapeutic option against MBL-producing carbapenem-resistant *P. aeruginosa*. Among the combinations evaluated, cefiderocol/zidebactam was the most effective therapeutic option, supported by the potent inhibitory activity of zidebactam against PAC-1. Xeruborbactam also demonstrated significant, albeit lower, inhibitory activity against PAC-1 compared with zidebactam, positioning the cefiderocol/xeruborbactam combination as an additional promising strategy warranting further preclinical evaluation. The detection of an identical Tn*1721*-like transposon in genetically unrelated *P. aeruginosa* STs underscores the dissemination potential of *bla*_PAC-1_, warranting continued epidemiological surveillance of PAC-type β-lactamases.

## References

[1] Kos VN, Déraspe M, McLaughlin RE et al The resistome of *Pseudomonas aeruginosa* in relationship to phenotypic susceptibility. Antimicrob Agents Chemother 2015; 59: 427–36. 10.1128/AAC.03954-14 25367914 PMC4291382

[2] Tada T, Shimada K, Satou K et al *Pseudomonas aeruginosa* clinical isolates in Nepal coproducing metallo-β-lactamases and 16S rRNA methyltransferases. Antimicrob Agents Chemother 2017; 61: e00694-17. 10.1128/AAC.00694-17 28696242 PMC5571325

[3] Bour M, Fournier D, Jové T et al Acquisition of class C β-lactamase PAC-1 by ST664 strains of *Pseudomonas aeruginosa*. Antimicrob Agents Chemother 2019; 63: e01375-19. 10.1128/AAC.01375-19 31527025 PMC6879236

[4] Teo JQ, Tang CY, Lim JC et al Genomic characterization of carbapenem-non-susceptible *Pseudomonas aeruginosa* in Singapore. Emerg Microbes Infect 2021; 10: 1706–16. 10.1080/22221751.2021.1968318 34384341 PMC8409972

[5] Zhong Y, Guo S, Thong S et al First report of environmental *bla*_PAC-1_-carrying *Aeromonas enteropelogenes*. Microbiol Spectr 2023; 11: e0139123. 10.1128/spectrum.01391-23 37909756 PMC10714797

[6] Tsai S, Nigo M, Kang D et al Cefepime-zidebactam therapy for extensively drug-resistant *Pseudomonas aeruginosa* and *Klebsiella pneumoniae* infection as a bridge to liver transplantation. JAC Antimicrob Resist 2025; 7: dlaf129. 10.1093/jacamr/dlaf129 40678583 PMC12268259

[7] Mancini S, Seth-Smith HMB, Kolesnik-Goldmann N et al Performance of disc diffusion and gradient strip test on different Mueller-Hinton agar media plates and ComASP^®^ and Bruker UMIC^®^ cefiderocol microdilution panels for cefiderocol susceptibility testing in carbapenem-resistant *Pseudomonas aeruginosa*. J Antimicrob Chemother 2025; 80: 2799–806. 10.1093/jac/dkaf301 40820354 PMC12494204

[8] Lysitsas M, Triantafillou E, Chatzipanagiotidou I et al Companion animals as reservoirs of multidrug resistance—a rare case of an XDR, NDM-1-producing *Pseudomonas aeruginosa* strain of feline origin in Greece. Vet Sci 2025; 12: 576. 10.3390/vetsci12060576 40559813 PMC12197500

[9] Saavedra S, Gutierrez MA, Duarte C. Comunicado técnico vigilancia intensificada por laboratorio de resistencia a ceftazidima/avibactam mediada por betalactamasas en Enterobacterales, en Colombia. Instituto Nacional de Salud, 2024. https://web.archive.org/web/20250429172532/http://www.ins.gov.co/BibliotecaDigital/comunicado-tecnico-vigilancia-intensificada-por-laboratorio-de-resistencia-a-ceftazidimaavibactam-mediada-por-betalactamasas-en-enterobacterales-en-colombia.pdf

[10] Castro BE, de la Cadena E, Escobar-Arcos JJ et al Emergence and dissemination of *bla*_KPC-31_ and *bla*_PAC-2_ among different species of Enterobacterales in Colombia: a new challenge for the microbiological laboratories. Microbiol Spectr 2025; 13: e0180525. 10.1128/spectrum.01805-25 41070989 PMC12584661

[11] Philippon A, Arlet G, Labia R et al Class C β-lactamases: molecular characteristics. Clin Microbiol Rev 2022; 35: e0015021. 10.1128/cmr.00150-21 35435729 PMC9491196

[12] West SE, Schweizer HP, Dall C et al Construction of improved *Escherichia-Pseudomonas* shuttle vectors derived from pUC18/19 and sequence of the region required for their replication in *Pseudomonas aeruginosa*. Gene 1994; 148: 81–6. 10.1016/0378-1119(94)90237-2 7926843

[13] EUCAST . Breakpoint tables for interpretation of MICs and zone diameters—16th edition. 2026. https://www.eucast.org/fileadmin/eucast/pdf/breakpoints/v_16.0_Breakpoint_Tables.pdf

[14] Berrow NS, Alderton D, Sainsbury S et al A versatile ligation-independent cloning method suitable for high-throughput expression screening applications. Nucleic Acids Res 2007; 35: e45. 10.1093/nar/gkm047 17317681 PMC1874605

[15] Hinchliffe P, González MM, Mojica MF et al Cross-class metallo-β-lactamase inhibition by bisthiazolidines reveals multiple binding modes. Proc Natl Acad Sci U S A 2016; 113: E3745–54. 10.1073/pnas.1601368113 27303030 PMC4932952

[16] Ibuka AS, Ishii Y, Galleni M et al Crystal structure of extended-spectrum β-lactamase Toho-1: insights into the molecular mechanism for catalytic reaction and substrate specificity expansion. Biochemistry 2003; 42: 10634–43. 10.1021/bi0342822 12962487

[17] Le Terrier C, Mlynarcik P, Sadek M et al Relative inhibitory activities of newly developed diazabicyclooctanes, boronic acid derivatives, and penicillin-based sulfone β-lactamase inhibitors against broad-spectrum AmpC β-lactamases. Antimicrob Agents Chemother 2024; 68: e0077524. 10.1128/aac.00775-24 39365068 PMC11539244

[18] Wick RR, Judd LM, Gorrie CL et al Unicycler: resolving bacterial genome assemblies from short and long sequencing reads. PLoS Comput Biol 2017; 13: e1005595. 10.1371/journal.pcbi.1005595 28594827 PMC5481147

[19] Moya B, Barcelo IM, Bhagwat S et al WCK 5107 (Zidebactam) and WCK 5153 are novel inhibitors of PBP2 showing potent “β-lactam enhancer” activity against *Pseudomonas aeruginosa*, including multidrug-resistant metallo-β-lactamase-producing high-risk clones. Antimicrob Agents Chemother 2017; 61: e02529-16. 10.1128/AAC.02529-16 28289035 PMC5444176

